# Design of New Polyacrylate Microcapsules to Modify the Water-Soluble Active Substances Release

**DOI:** 10.3390/polym13050809

**Published:** 2021-03-06

**Authors:** Valentina Sabatini, Laura Pellicano, Hermes Farina, Eleonora Pargoletti, Luisa Annunziata, Marco A. Ortenzi, Alessandro Stori, Giuseppe Cappelletti

**Affiliations:** 1 Dipartimento di Chimica, Università degli Studi di Milano, Via Golgi 19, 20133 Milan, Italy; vsabatini@outlook.com (V.S.); laura.pellicano@studenti.unimi.it (L.P.); hermes.farina@unimi.it (H.F.); eleonora.pargoletti@unimi.it (E.P.); marco.ortenzi@unimi.it (M.A.O.); 2 Consorzio Interuniversitario per la Scienza e Tecnologia dei Materiali (INSTM), Via Giusti 9, 50121 Firenze, Italy; luisa.annunziata@unimi.it; 3 CRC Materiali Polimerici “LaMPo”, Dipartimento di Chimica, Università degli Studi di Milano, Via Golgi 19, 20133 Milano, Italy; 4 AMVIC srl, Piazza Santo Stefano 6, 20122 Milano, Italy; alessandro.stori@icloud.com

**Keywords:** polyacrylate, water-based latex, Pickering emulsion, microencapsulation, controlled release

## Abstract

Despite the poor photochemical stability of capsules walls, polyacrylate is one of the most successful polymers for microencapsulation. To improve polyacrylate performance, the combined use of different acrylate-based polymers could be exploited. Herein butyl methacrylate (BUMA)-based lattices were obtained via free radical polymerization in water by adding (i) methacrylic acid (MA)/methyl methacrylate (MMA) and (ii) methacrylamide (MAC) respectively, as an aqueous phase in Pickering emulsions, thanks to both the excellent polymer shells’ stability and the high encapsulation efficiency. A series of BUMA_MA_MMA terpolymers with complex macromolecular structures and BUMA_MAC linear copolymers were synthesized and used as dispersing media of an active material. Rate and yield of encapsulation, active substance adsorption onto the polymer wall, capsule morphology, shelf-life and controlled release were investigated. The effectiveness of the prepared BUMA-based microcapsules was demonstrated: BUMA-based terpolymers together with the modified ones (BUMA_MAC) led to slow (within ca. 60 h) and fast (in around 10 h) releasing microcapsules, respectively.

## 1. Introduction

In the last decade, microencapsulation technique of effective liquid and solid agents has attracted wide interest from several industrial sectors, such as the food chemistry, building, health and beauty industries [1,2,3,4]. The advantages of such a procedure are manifold: for example, it can be used to protect active materials, such as probiotics and drugs, during their passage through animals and human bodies [5] or in the construction industry, for the protection of change phase and thermal energy storage materials [6]. Moreover, encapsulation allows several fertilizers and agricultural handlings to be released over time [7]. The key to the success of every microencapsulation process is the formation of a resistant and continuous protective envelope for the entrapped active substance and keep it safe from the surrounding environment. Methods and materials adopted for the encapsulation process contribute to define microcapsules properties and kinetic release. In particular, the methods commonly available for the formulation of microcapsules can be divided into physical and chemical approaches [8]. Both these methods confer some useful properties depending on the desired characteristics of the microcapsules produced. These include microcapsules’ shape and size, shells thickness and mechanical resistance [9]. Furthermore, the selection of the most suitable industrial production technique depends on multiple process parameters, such as the physical state of the host material (liquid or solid, hydrophilic or hydrophobic, and so on), the inorganic or organic nature, the miscibility as well as the chemical compatibility between envelope and active agent [10,11,12,13]. The most reported physical techniques are pan coating [14] and spray drying [15] due to their easy preparation technology that favors fast and cheap lab-to-industrial scale productions [16]. However, particles of high granulometry (often with average sizes larger than 1 mm) and not uniformly coated microcapsules have been reported as part of such processes, respectively [17]. On the other side, chemical microencapsulation techniques are strictly based on chemical interactions between the different materials used. These chemical interactions result in polymerization of several substances that form the wall of microcapsules. The most common types of chemical technique used are the interfacial polymerization [18] and in situ polymerization [19]. Although chemical methods for microencapsulation are very popular thanks to the possibility to tailor the final properties during the synthesis of protective materials and for this purpose have been used widely in many industrial sectors, they have several drawbacks. Indeed, to maintain some operative conditions, strong solvents and acids are required [20]. Moreover, polymerization catalysts are usually toxic [21]: for example, chemical scavengers can be used to reduce the amount of free unreacted harmful chemicals, but this increases production costs [22]. Moreover, both chemical methods are known to be very successful in producing microcapsules with diameters lower than 100 microns. The smaller their size the larger the volume fraction that would be necessary to deliver the desirable results and thus, the poor margin of tolerance of the obtainable microcapsules with different dimensions raise skepticism over the use of chemical methods to produce them [22].

In this scenario, the combination of physical and chemical methods is an emerging approach, thanks to the hopeful benefits deriving from each of these techniques and the minimization of the already cited drawbacks. The physicochemical methods are mainly based on the formation of walls from preformed polymers, and among the most used methods, ionic gelation [23], complex coacervation [24] and Pickering emulsion [25] can be found. Ionic gelation produces generally very porous and permeable hydrogels [26], and this can be beneficial in some applications, such as in flavors delivery [27] but in others, such as building, can be problematic [28]. Complex coacervation can produce a wide range of microcapsules dimension, from 10 to 1000 microns, and utilizes environmentally friendly materials, but sometimes the yield of production is very poor depending on the type of emulsifier chosen [29]. Moreover, in this plethora of methods, Pickering emulsion is the process during which, for example in a water-in-oil emulsion, micro-sized droplets of water are dispersed in a continuous oil phase and polymer nanoparticles are located at the interface. Depending on their surface tension, these nanoparticles can stabilize the emulsion in the colloidal sense [30]. Nagayama et al. [31] were the first to demonstrate that Pickering emulsions can be transformed into microcapsules when the interfacial nanoparticles are either linked or fused together to form a continuous wall. The greatest advantage of such kind of procedure is that it allows process adjustments during the wall formation step. This in-process intervention cannot be done for the other techniques discussed above. On the other side, the choice of microcapsules “building” materials draws the morphological properties of the resulting protective shells, such as permeability and release performances. For instance, biopolymers such as alginates and polysaccharides favor the development of microcapsules with tailored surface features [32,33]. Moreover, the use of biopolymers is useful for applications involving the biodegradable nature of the protective shells, for example in the case of zootechnic probiotics [34]. However, the use of synthetic polymers as encapsulating materials is of particular interest in the field of porous and permeable microcapsules preparation [35] and, in this latter case, polyacrylates are the main protagonists [36], due to their easy and cheap preparation using both solvent and water-based dispersing media [37,38,39], the possibility to use ad hoc functionalized monomers to obtain smart shells [40] and, last but not least, the biocompatible and biodegradable nature of some acrylic monomers, such as acrylamide [41]. Thus, the fine modulation of pores dimension and distribution are the key properties of such enveloping materials [42].

Nowadays, in the field of microcapsules preparation processes, one of the main issues to overcome is to obtain a stable product during storage with consistent encapsulation and release efficiency. The latter demand is quite difficult to reach because the common materials used for lattices formulation, e.g., alginates [43], polyacrylates [44], pectin/casein blends [45,46] and gelatines [47], are very sensitive to processing and environmental factors, such as temperature, solar irradiation, humidity and pH variation [48], and this results in poor encapsulation efficacy, reduced microcapsules shelf-life and uncontrolled release kinetics. Vincent et al. [49] reported the preparation of butyl methacrylate–methacrylic acid (BUMA_MA) latex for the microencapsulation and release process of different microorganisms as pesticide agents. Despite the comparable pesticide behaviour of their latex with common chemical products, they described that during emulsion process upon adding ethanol as co-solvent, the latex particles within the aqueous droplets are colloidally unstable leading to coalescence and resulting in a poor microcapsules formation. This behaviour should be due to the high affinity of MA hydroxyl groups with water (latex dispersing medium), that obstructs the formation of polymer particles at the interphase between water and oil phases [50].

To the best of the authors’ knowledge, few studies in the literature about microcapsules formation from Pickering emulsion procedure, tailoring different polyacrylate-based polymers, have been reported so far. Hence, in this work, we prepared novel polyacrylic lattices (i.e., a series of BUMA-based terpolymers with complex macromolecular structure and BUMA_methacrylamide (MAC) linear copolymers) as protective materials for the microencapsulation of an active agent, by water-based free radical polymerization procedure during Pickering emulsion. These microcapsules were designed to: (i) be formulated exploiting environmentally friendly materials to achieve more biodegradable polymeric matter; (ii) preserve the active material from the surrounding environment; (iii) tune the effective agent release; and (iv) be re-dispersed in water to form a free-flowing non-viscous aqueous formulation. Therefore, a deep characterization of the prepared polymeric latex, via ^1^H nuclear magnetic resonance (NMR), Fourier-transform infrared and thermal analyses, and of microcapsules through dynamic light scattering and scanning electron microscopy studies, was conducted and is discussed here as well as the corresponding kinetics of active material release.

## 2. Materials and Methods

### 2.1. Materials

N-butyl methacrylate (BUMA, 99%), methacrylic acid (MA, 99%), methyl methacrylate (MMA, 99%), pentaerythritol triacrylate (T3, technical grade), methacrylamide (MAC, 98%), sodium persulfate (Na_2_S_2_O_8_, ≥99.9%), sodium dodecyl sulfate (SDS, ≥99.9%), trifluoroacetic acid-d (C_2_DF_3_O_2_, 99.5 atom % D), sunflower oil, sodium chloride (NaCl, ≥99.0%), iron (II, III) oxide nanopowder (Fe_3_O_4_, 50–100 nm particle size), ethanol (96%), methyl orange (MO), KNO_3_ 10^−2^ M and distilled water Chromasolv^®^ (≥99.9%) were supplied by Sigma Aldrich (Milan, Italy) and used without further purification.

### 2.2. Synthesis of Butyl Methacrylate–Methacrylic Acid (BUMA_MA), BUMA_MA_Methyl Methacrylate (MMA), BUMA_MA_MMA_Pentaerythritol Triacrylate (T3) and BUMA_Methacrylamide (MAC) Copolymers

BUMA_MA copolymer (used as reference), two terpolymers, i.e., BUMA_MA_MMA and BUMA_MA_MMA_T3, and BUMA_MAC copolymers were synthesized via free radical polymerization. Table 1 and Table S1 show the molar ratio and the exact amount of the monomers used for the syntheses, respectively.

In a typical polymerization procedure, a 250 cm^3^ three-necked round bottom flask was equipped with a reflux condenser having a nitrogen inlet adapter, an internal thermometer adapter and a mechanical stirrer. The flask was flushed with nitrogen, charged with 100 cm^3^ of distilled water, 0.5 g of sodium dodecyl sulfate (SDS) used as latex stabilizer [49], Na_2_S_2_O_8_ (mol Na_2_S_2_O_8_ = 1% mol mol^−1^_∑acrylate monomers_) and acrylate monomers, according to the desired polymer. The polymerization mixture was put in an oil bath, mechanically stirred at around 280–300 rpm, heated for 24 h at 69 °C and then gradually cooled down to room temperature. The reaction yields, determined gravimetrically, were around 100% for all the investigated systems.

### 2.3. Polymer Latex Characterization: Fourier Transform Infrared (FT-IR), Nuclear Magnetic Resonance (^1^H NMR), Dynamic Light Scattering (DLS) and Differential Scanning Calorimetry (DSC) Analyses

For spectroscopic and thermal characterizations, an aliquot of the lattices prepared was previously dried under N_2_ at 50 °C for 24 h.

Fourier transform-infrared (FT-IR) spectra were obtained on a Spectrum 100 spectrophotometer (Perkin Elmer) in attenuated total reflection (ATR) mode using a resolution of 4.0 and 256 scans, in a range of wavenumbers between 4000 and 450 cm^−1^. A single-bounce diamond crystal was used with an incidence angle of 45°. ^1^H NMR spectra were collected at 25 °C with a BRUKER 400 MHz spectrometer. All samples were prepared dissolving 8–10 mg of polymer in 1 cm^3^ of C_2_DF_3_O_2_.

Dynamic light scattering (DLS) analyses were carried out to study the lattices particles size distribution. Samples were analysed after a 1:100 dilution in KNO_3_ 10^−2^ M to adjust the ionic strength (this step does not affect the correctness of the results obtained, as already reported in the literature) [51,52], using a Malvern Zetasizer NANO ZS (at 25 °C; Malvern Panalytical, Malvern). Measurements were performed on two different aliquots, for 30 scans each. 

Differential scanning calorimetry (DSC) analyses were conducted using a Mettler Toledo DSC1; the analyses were conducted weighting 5–10 mg of each sample in a standard 40 µL aluminium pan, using the following temperature cycles:

1. heating from 0 °C to 100 °C at 20 °C min^−1^;
2. 2 min isotherm at 100 °C;
3. cooling from 100 °C to 0 °C at 20 °C min^−1^;
4. 2 min isotherm at 0 °C;
5. heating from 0 °C to 150 °C at 20 °C min^−1^.

using an empty 40 µL aluminium pan as reference.

### 2.4. Preparation Process for Methyl Orange (MO) Encapsulation within Polyacrylates Microcapsules and Scanning Electron Microscopy (SEM) Characterization

**Scheme 1:**
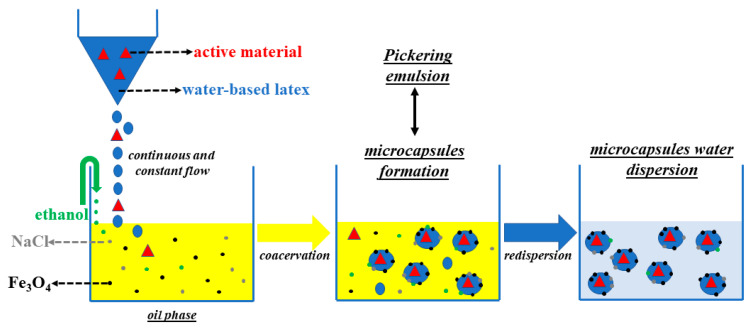
Schematic representation of methyl orange (MO) encapsulation within polyacrylate microcapsules.

In a 300 cm^3^ glass jacket reactor 88 cm^3^ of sunflower oil, 224 mg of Fe_3_O_4_ and 721 mg of NaCl were added. The mixture was processed with a mechanical stirrer (around 380–400 rpm) for 15 min at room temperature. In a separate tube, 1 cm^3^ of a methyl orange (MO) solution with a concentration of 600 ppm was added to 16 cm^3^ of the as-synthesized latex (see Scheme 1). Then, 6 cm^3^ of ethanol and the solution of latex/methyl orange were added dropwise and the mixture was mechanically stirred (around 250–300 rpm) for 20–30 min at room temperature. The mixture was decanted for 2 h in order to separate the yellowish microcapsules from the supernatant oil phase. The oil phase was removed, and the resulting microcapsules were re-dispersed in water. The process was repeated until oil was no longer observable. 

An aliquot of the microcapsules obtained was dried for 2 h at room temperature on a blotting paper in order to perform scanning electron microscopy (SEM) images. This analysis was carried out using a SEM Hitachi TM-1000 (Tokio, Japan).

### 2.5. MO Kinetic Release

To deeply investigate the encapsulation and release capacities, methyl orange was chosen to make easier the correct evaluation through ultraviolet/visible (UV/Vis) spectroscopy. Specifically, once the MO-encapsulated microcapsules were obtained, 1 g of them were dispersed into 100 cm^3^ of distilled water (pH 5.5) under magnetic stirring (ω = 50–80 rpm). The absorbance spectra (recorded in the range between 650 and 350 nm by using SHIMADZU UV 2600 spectrophotometer (Kyoto, Japan)) of each sampling (1.5 cm^3^) were acquired at regular time intervals for a total of 120 h, in order to monitor the release of MO over time. Moreover, to compute the exact MO amount, a calibration curve for MO in water was obtained at pH 5.5 (see Figure S1), following the absorbance value at the maximum wavelength of 465 nm and obtaining a molar extinction coefficient (ε) equal to (25,320 ± 70) dm^3^ mol^−1^ cm^−1^. The encapsulation efficiency percentage (EE%) was computed according to [53].

## 3. Results and Discussion

### 3.1. Synthesis and Characterization of BUMA-Based Lattices

Several approaches have been already reported in literature to improve microcapsules formation either using hydroxyl functionalized monomers, such as MA neutralized by CaCl_2_ or NaOH, or by adding emulsification agents [54]. However, the difficulty in reaching a complete salt-based protection of hydroxyl groups and the possible interference between process additives and active substances, result in lattices that are not stable with the formation of flocculation by-products [55]. Herein, to improve the performances of polyacrylic lattices, a novel BUMA-based terpolymer adding MMA as comonomer to BUMA and MA was synthesized. In this way, a decrease in the affinity between the polymer latex and the aqueous dispersing media, thanks to the presence of apolar -CH_3_ side groups instead of hydroxyl functionalities, was attained. Specifically, two new kinds of BUMA-based polymers were obtained via free radical polymerization in water by adding (i) MA/MMA as comonomers plus T3, as branching agent (adopting MA/BUMA molar ratio of 0.25% mol/mol for both BUMA_MA_MMA samples) and (ii) MAC, improving both the polymer shells’ stability and the encapsulation efficiency. As a reference, BUMA_MA latex (having MA/BUMA molar ratio of 0.83 mol mol^−1^) was prepared as already described in literature [49]. 

**Figure 1:**
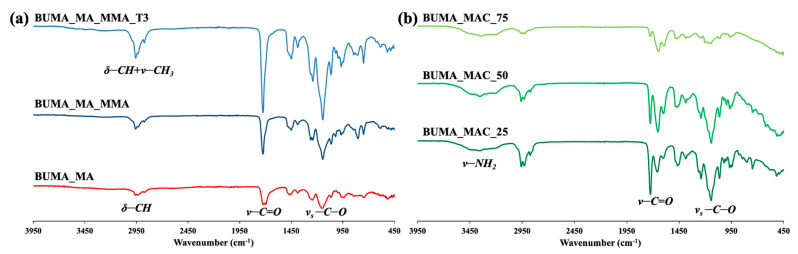
Fourier transform infrared (FT-IR) spectra of (**a**) BUMA_MA and BUMA-based terpolymers and (**b**) BUMA_MAC copolymers.

Figure 1a shows the comparison between BUMA_MA, BUMA_MA_MMA and BUMA_MA_MMA_T3 FT-IR spectra. Notably, it is possible to appreciate several peaks related to: the bending of C–H aliphatic bonds at ~3100–2800 cm^−1^ (*δ-CH*), the stretching of carbonyl ester groups -C=O in the range between ~1750 cm^−1^ and ~1600 cm^−1^ (*v-C=O*), and the characteristic absorption band for the symmetric stretching vibration of -C–O conjugated to carbonyl ester groups (1350–1100 cm^−1^) (*v_s_–C–O*) [56]. In the case of MMA-based samples, the stretching of –CH_3_ groups conjugated to carbonyl functionalities is also detectable in the range ~3100–2800 cm^−1^ (*v–CH*_3_) [57]. As expected, the BUMA_MA sample, having a relatively high content of methacrylic acid, shows the presence of the broad band related to –OH stretching above 3000 cm^−1^ and a double C=O peak due to the simultaneous presence of ester and acid moieties along the macromolecular chain.

**Figure 2:**
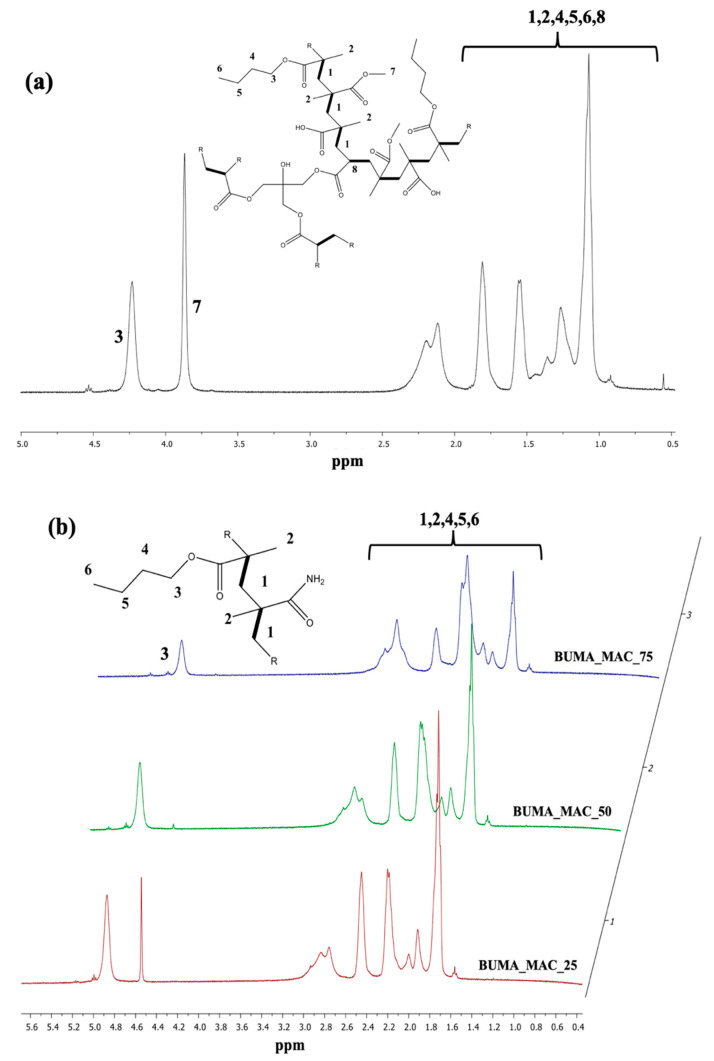
Nuclear magnetic resonance (^1^H NMR) spectra of (**a**) BUMA_MA_MMA_T3 and (**b**) BUMA_MAC copolymers.

Since T3 is present in very small quantities, no significant differences can be observed between BUMA_MA_MMA and BUMA_MA_MMA_T3 samples. As discussed by Mallo et al. [58], the use of branching agents can be a way to obtain non-linear polymer lattices, which are insoluble in water in the form of three-dimensional networks but, at the same time tend to swell. As such, the polymeric shell can form at the interphase between water and oil phases during the emulsion process. Thus, to further reduce the affinity between latex and water, a novel terpolymer with complex macromolecular structure was developed (namely BUMA_MA_MMA_T3) by adding 0.5% mol/mol of pentaerythritol triacrylate, T3, as branching agent. Such a low concentration of T3, i.e., from 0.05% to 1.0% mol mol^−1^, is mandatory to avoid the flocculation of parts of polymer from water media, as also confirmed by literature data [58]. Figure 2a shows the BUMA_MA_MMA_T3 ^1^H NMR spectrum, where the presence of the branching agent cannot be highlighted due to its exiguous amount. For clarity, the reference and BUMA_MA_MMA ^1^H NMR spectra have been compared to the one relative to BUMA_MA_MMA_T3 sample (Figure S2). 

Furthermore, since our goals are to increase the microcapsules stability and to modulate the active substance (herein MO) encapsulation and its release, other kinds of polyacrylate-based material were investigated. As reported by Stranimaier et al. [59], amide functional monomers are useful for the preparation of waterborne lattices due to their apolar nature that favors: (i) the formation of polymer shells at water/oil interphase, the improved (ii) photochemical resistance and (iii) stable appearance of the corresponding microcapsules. Moreover, alongside these promising features, acrylamide-based materials are susceptible to different biodegradation processes [60]. Hence, methacrylamide (MAC) was selected as comonomer in combination with BUMA in order to synthesize new lattices with improved photochemical stability and a potential biodegradable behavior. As summarized in Table 1, a series of BUMA_MAC copolymers were prepared exploiting different molar ratios between the two monomers adopted. Figure 1b reports the comparison between BUMA_MAC_25, BUMA_MAC_50 and BUMA_MAC_75. The presence of -NH_2_ moieties is clearly visible due to the broad signal at 3600–3200 cm^−1^ related to the stretching of NH_2_ group, that decreases as the quantity of MAC gets lower in the copolymers. The same holds true for the double C=O peak in the 1750–1600 cm^−1^ region, with the peak at higher frequencies relative to the ester moiety of BUMA and the one at lower frequencies of the amide moiety of MAC. Moreover, the comparison among the ^1^H NMR spectra reported in Figure 2b highlights the decrease of NMR peak “**3**” relative to BUMA height and width as the amount of MAC gets higher. 

To unravel the stability of the as-prepared lattices, DLS measurements and DSC analyses were performed. At first, Table 2 (2nd column) and Figure S3 show the data obtained relative to DLS measurements, indicating the average particles diameter for each copolymer. By comparing the same polymers family, we can assess that BUMA_MA_MMA_T3 latex seems to be the most performing one, since the polymer particles have a single average dimension of about 80 ± 20 nm, lower than the BUMA_MA (180 ± 70 nm) and BUMA_MA_MMA (two populations: 85 ± 15 and 250 ± 60 nm) ones.

It is worth noting that in the latter case, the addition of only an apolar monomer, i.e., MMA, is not enough to ensure the formation of homogenous and stable latex, since polymeric aggregates of ca. 250 nm can be formed. Conversely, concerning the BUMA_MAC family, BUMA_MAC_75 latex seems to be the most stable one and DLS measurements reveal the presence of particles with a very small hydrodynamic diameter of around 12 ± 3 nm.

Lastly, during Pickering emulsions a latex can be transformed into microcapsules when the interfacial nanoparticles are either linked or fused together to form a continuous wall. This step, usually called “locking”, can be achieved via several approaches, such as chemical cross-linking, in situ polymerization, thermal softening and solvent-instability of the particles [61]. However, when nanoparticles are polymeric, a continuous wall can thus be formed around the aqueous droplet, leading to the formation of a microcapsule. The glass transition temperature, *T_g_*, of a polymer is a key value for the occurrence of the locking phenomena, since it corresponds to the temperature at which a polymer switches from hard and glassy form to soft and viscous one. In particular, *T_g_* values higher than room temperature favour the fusion of the latex polymer particles in a continuous polymeric shell and, therefore, promote high microcapsules yield [62]. 

Herein, according to Table 1, the molar ratio between monomers was established via the Fox equation (Equation (1)) [63] in order to ensure, during the Pickering emulsion process, the formation of stable and consistent polymer walls and therefore favour the locking process:

1/*T_g_* = w_1_/*T_g_*_,1_ + *w_n_*/*T_g_*_,n_ (1)

where *T_g_* corresponds to the theoretical glass transition temperature value of the resultant copolymer or terpolymer; *w*_1_ and *w_n_* are the weight fractions of each component; *T_g_*_,1_ and *T*_*g*,*n*_ are the glass transition temperatures of BUMA, MA, MMA and MAC homopolymers, 293–501–378–486 K, respectively. Glass transitions were determined during the first heating scan (see Figure S4). Notably, from the comparison of data reported in the 3rd and 4th columns of Table 2, the *T_g_* values of our samples are not in good agreement with theoretical values and in some cases cannot be detected; this might be due to the presence of sodium salts in the copolymers containing MA, and to the water uptake of –CONH_2_ moieties in copolymers containing MAC. Nevertheless, *T_g_* is always higher than room temperature.

### 3.2. Pickering Emulsions and MO Kinetic Release of BUMA_MA_MMA_T3 and BUMA_MAC_75 Lattices

Once the lattices of BUMA_MA, BUMA_MA_MMA, BUMA_MA_MMA_T3 and BUMA_MAC copolymers were prepared, several Pickering emulsions, according to the procedure summarized in Scheme 1, were formulated. NaCl and iron oxide nanoparticles were added in the oil phase respectively to stabilize the emulsion against Ostwald ripening in the coacervation step and to serve as light absorbing material in the final formulation [62]. Upon mixing the water-based latex and the oil phase, a Pickering water-in-oil emulsion was obtained. The latex nanoparticles have a surface energy which is intermediate between oil and water, thus they position themselves at the interface between water and oil. With the addition of ethanol as co-solvent [64], the latex particles form a continuous wall of polymer around the aqueous droplets, that results in the formation of insoluble microcapsules (MC). Remarkably, the fabrication of these capsules does not require any organic solvent and is entirely performed at room temperature. According to Vincent et al. [49], after the addition of ethanol, the latex particles of BUMA_MA reference collapsed and coalesced, resulting in poor microcapsule formation yield. Despite the addition of MMA monomer, BUMA_MA_MMA latex also showed the same behaviour, i.e., an unsatisfactory conversion of latex particles in polymer capsules. On the other hand, it is worth noting both the successful process of microcapsules formation and the MO encapsulation for BUMA_MA_MMA_T3 latex, thanks to the combined used of apolar and branching monomers that allow the formation of a cross-linked latex. Lastly, the series of BUMA_MAC copolymers were also tested in the Pickering emulsion process but only the sample with the higher amount of MAC, namely BUMA_MAC_75, resulted in promising microcapsules formation. The behaviour of BUMA_MAC_25 and BUMA_MAC_50 samples is probably due to the low amount of MAC monomer that was not enough to promote the formation of polymer shells at the water/oil interphase [65].

**Figure 3:**
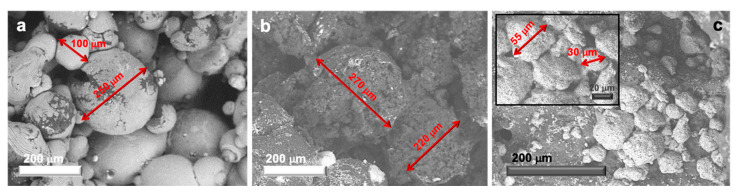
Scanning electron microscopy (SEM) micrographs displaying the actual capsules size (highlighted with red arrows) relative to: (**a**) MC(BUMA_MA), (**b**) MC(BUMA_MA_MMA_T3) and (**c**) MC(BUMA_MAC_75) (inset: high magnification image).

Hence, considering the optimal microcapsules formation obtained with our novel BUMA_MA_MMA_T3 and BUMA_MAC_75 lattices, both microscopy analyses and kinetic tests were carried out to investigate these systems in depth. In particular, the trend in the DLS lattices particles size was also corroborated by the aspect and dimension of the microcapsules, observed by SEM. Indeed, as regards, BUMA_MA_MMA_T3-based microcapsules (MC(BUMA_MA_MMA_T3)), they show a rough surface with an average diameter of 220–270 μm, as clearly visible in Figure 3b. This may suggest the formation of a continuous polymer wall probably characterized by the presence of pores large enough to allow the passage of the active substance contained in the MC core, thus favouring its release [66,67]. 

On the other hand, the reference BUMA_MA latex gave rise to microcapsules with both an average diameter comparable with the one of MC(BUMA_MA_MMA_T3) and, even, smaller (ca. 100 μm; Figure 3a), but characterized by a very smooth surface. By contrast, MC(BUMA_MAC_75) exhibits a very small average diameter of about 30–55 μm (Figure 3c), with a quite rough external surface. Hence, we can infer that the difference in both microcapsules dimensions and surface porosity can suggest a different release behavior of the same active substance, i.e., MO. For this reason, kinetic tests were performed by using the best performing samples (namely, MC(BUMA_MA_MMA_T3) and MC(BUMA_MAC_75)) following the MO release over time by UV/Vis spectroscopy.

**Figure 4:**
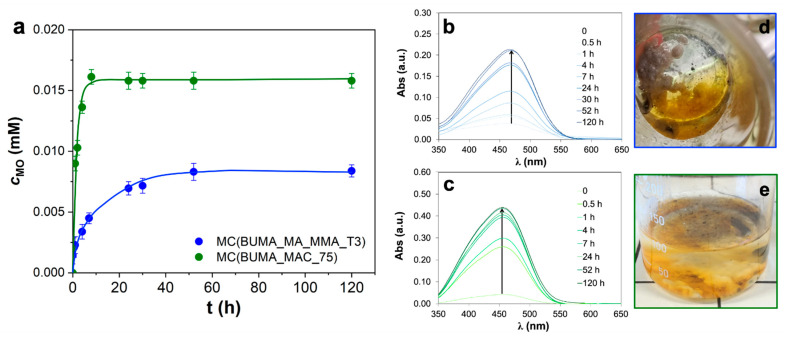
(**a**) Released MO concentration curves relative to MC(BUMA_MA_MMA_T3) and MC(BUMA_MAC_75) systems. Error bars computed on three different replicates. (**b**,**c**) Corresponding kinetics followed through ultraviolet/visible (UV/Vis) analysis, along with the relative (**d**,**e**) photos taken at the end of the test (120 h).

Figure 4a clearly shows the released dye concentration up to 120 h, alongside with the corresponding spectroscopic data (Figure 4b,c) and photos displaying the actual presence of MO in the aqueous medium surrounding the microcapsules at the end of the kinetics (Figure 4d,e). In particular, with the bigger MC(BUMA_MA_MMA_T3) system, a partial release was achieved only after 60 h (Figure 4a,b; encapsulation efficiency percentage, EE% of 46%), thus revealing a slower kinetic with respect to the one relative to MC(BUMA_MAC_75). Indeed, for the latter, the curve trend at the beginning is very sharp leading to an almost full MO release already after 10 h (EE% ca. 86%; see Figure 4a,c). However, a possible MO adsorption together with a direct encapsulation cannot be excluded. Actually, the different release behavior of the aforementioned microcapsules could be rather complex depending on (i) the adsorption/encapsulation features; (ii) the chemical affinity of the active substance towards the polymer wall (having different polarity and macromolecular structure); (iii) the porosity of the microcapsules and the relative size. Notably, in the present case, it seems that mainly the porosity and the size of the microcapsules play a pivotal role in affecting the release features, being MC(BUMA_MAC_75) three-fold smaller than MC(BUMA_MA_MMA_T3).

Hence we successfully demonstrated that, by tailoring the polymeric materials used in the Pickering emulsions, it is possible to synthesize microcapsules with ad hoc morphological and surface features to be applied in different application fields, according to the desired releasing rate, and in particular, BUMA_MA_MMA_T3-based microcapsules appear to be the most effective for the slow release process of MO; on the other hand, BUMA_MAC shells are performing as fast-releasing microcapsules.

## 4. Conclusions

In the field of microencapsulation, the need to develop new polymer lattices, characterized by improved shelf-life and high encapsulation-release efficiency, is always pressing due to the wide plethora of industrial applications involved. In this context, the use of polyacrylates, bearing apolar, cross-linkable and potential biodegradable monomers along the polymeric chains, could be a way to create tailor-made microcapsules with permeable surfaces and controlled kinetics release. 

Herein, butyl methacrylate (BUMA), methacrylic acid (MA) and methyl methacrylate (MMA) latex was synthesized via free radical polymerization in a water media using just 0.5% mol mol^−1^ of a branching monomer, pentaerythritol triacrylate (T3). Furthermore, a series of BUMA_methacrylamide (MAC) copolymers were prepared, varying the molar percentage of MAC monomer (25–50–75% mol mol^−1^). At first, the macromolecular structures, thermal features and hydrodynamic volumes were investigated before and after the Pickering emulsion, i.e., the microencapsulation process used here; moreover, the morphology of the microcapsules obtained was assessed via scanning electron microscopy, demonstrating the formation of polymer shells with average diameters of 270 and 55 μm in the case of BUMA_MA_MMA_T3 and BUMA_MAC_75, respectively.

The microcapsules obtained were studied by kinetic release, focusing on rate of release and physical resistance of the microcapsules dispersed in water. Both polymeric lattices showed very promising features as protective envelopes. Notably, BUMA_MA_MMA_T3 latex seems to be the most suitable one in the case of slow release, due to its ability to form bigger microcapsules and thus protect for longer times the active agents entrapped from the surrounding environment. Conversely, for BUMA_MAC_75-based microcapsules fast kinetic release was observed. Hence, to the best of the authors’ knowledge the synthesis of such kinds of lattice and their use in Pickering emulsion for microencapsulation process of effective agents has not been reported so far. Specifically, the present research has led to an in depth comprehension of microcapsules formation and kinetic release of BUMA-based lattices that can be successfully adopted as protective polymer shells for the controlled delivery of active materials.

## Figures and Tables

**Table 1:** Molar ratio of reagents adopted for the synthesis of butyl methacrylate (BUMA)-based polymers. MA = methacrylic acid; BUMA = n-butyl methacrylate; MMA = methyl methacrylate; T3 = pentaerythritol triacrylate; MAC = methacrylamide.

Sample	MA/BUMA (mol mol^−1^)	MMA/BUMA (mol mol^−1^)	T3/BUMA (mol mol^−1^)	MAC/BUMA (mol mol^−1^)
BUMA_MA	0.83	-	-	-
BUMA_MA_MMA	0.25	0.75	-	-
BUMA_MA_MMA_T3	0.25	0.75	0.005	-
BUMA_MAC_25	-	-	-	0.33
BUMA_MAC_50	-	-	-	1.00
BUMA_MAC_75	-	-	-	3.00

**Table 2:** Molar ratio of reagents adopted for the synthesis of BUMA-based polymers.

Sample	<d^DLS^> (nm)	Fox *T_g_* (°C)	Real *T_g_* (°C)
BUMA_MA	180 ± 70	67.1	n.d.
BUMA_MA_MMA	(85 ± 15); (250 ± 60)	55.5	78.2
BUMA_MA_MMA_T3	80 ± 20	55.5	75.2
BUMA_MAC_25	(37 ± 7); (170 ± 30)	40.8	46.1
BUMA_MAC_50	(25 ± 4); (80 ± 10)	71.1	51.0
BUMA_MAC_75	12 ± 3	120.3	n.d.

## Data Availability

Data are contained within the article or supplementary material.

## Supplementary Materials

The following are available online at https://www.mdpi.com/2073-4360/13/5/809/s1, Table S1: amounts of the reagents adopted for the synthesis of BUMA-based polymers; Figure S1: (a) methyl orange UV/Vis spectra at different molecule concentrations, (b) relative calibration plot at wavelength fixed at 465 nm; Figure S2: ^1^H NMR spectrum of (a) BUMA_MA and (b) BUMA_MA_MMA polymers; Figure S3: Dynamic light scattering data relative to BUMA_MA, BUMA_MA_MMA, BUMA_MA_MMA_T3 and BUMA_MAC_75 systems; Figure S4: DSC curves relative to (a) BUMA_MA, (b) BUMA_MA_MMA, (c) BUMA_MA_MMA_T3, (d) BUMA_MAC_25, (e) BUMA_MAC_50 and (f) BUMA_MAC_75.
