# Supplementary material for: Design of New Polyacrylate Microcapsules to Modify the Water-Soluble Active Substances Release
Source: Polymers (Basel). 2021 Mar 6;13(5):809. doi: 10.3390/polym13050809 (PMC7961822; doi:10.3390/polym13050809)
Supplement: Supplementary file 1 [file polymers-13-00809-s001.pdf]

polymers

MDPI

Supporting Information

Design of New Polyacrylate Microcapsules to Modify the Water-soluble Active Substances Release

V. Sabatini [1,2], L. Pellicano [1], H. Farina [1,2,3], E. Pargoletti [1,2], L. Annunziata [2,3], M.A. Ortenzi [1,2,3], A. Stori [4] and G. Cappelletti [1,2,3,*]

[1] Dipartimento di Chimica, Università degli Studi di Milano, Via Golgi 19, 20133 Milan, Italy; valentina.sabatini@unimi.it (V.S.), laura.pellicano@studenti.unimi.it (L.P.), hermes.farina@unimi.it (H.F.), eleonora.pargoletti@unimi.it (E.P.), marco.ortenzi@unimi.it (M.A.O.)
[2] Consorzio Interuniversitario per la Scienza e Tecnologia dei Materiali (INSTM), Via Giusti 9, 50121, Firenze, Italy
[3] CRC Materiali Polimerici "LaMPo", Dipartimento di Chimica, Università degli Studi di Milano, Via Golgi 19, 20133 Milano, Italy; luisa.annunziata@unimi.it (L.A.)
[4] AMVIC srl, Piazza Santo Stefano 6, 20122, Milano, Italy; alessandro.stori@icloud.com (A.S.)
* Correspondence: giuseppe.cappelletti@unimi.it; Tel.: +39 0250314228 (G.C.)

Table S1. Amounts of the reagents adopted for the synthesis of BUMA-based polymers.

Sample	$Na_2S_2O_8$ (g)	BUMA (g)	MA (g)	MMA (g)	T_3 (g)	MAC (g)	SDS (g)
BUMA_MA	0.25	10.00	5.00	-	-	-	0.50
BUMA_MA_MMA	0.33	8.73	1.32	4.61	-	-	0.50
BUMA_MA_MMA_T3	0.30	8.73	1.32	4.60	0.10	-	0.50
BUMA_MAC_25	0.31	12.25	-	-	-	2.44	0.50
BUMA_MAC_50	0.35	9.17	-	-	-	5.49	0.50
BUMA_MAC_75	0.40	5.22	-	-	-	9.38	0.50

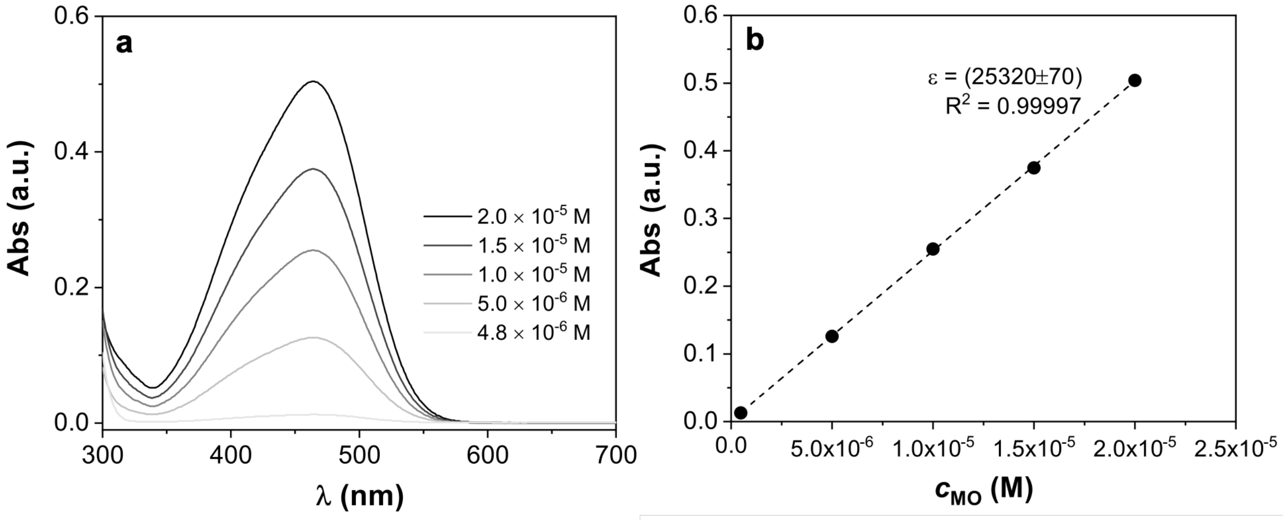

Figure S1. (a) Methyl orange UV/Vis spectra at different molecule concentrations. (b) Relative calibration plot at wavelength fixed at 465 nm. The corresponding molar extinction coefficient (ε) has been reported.

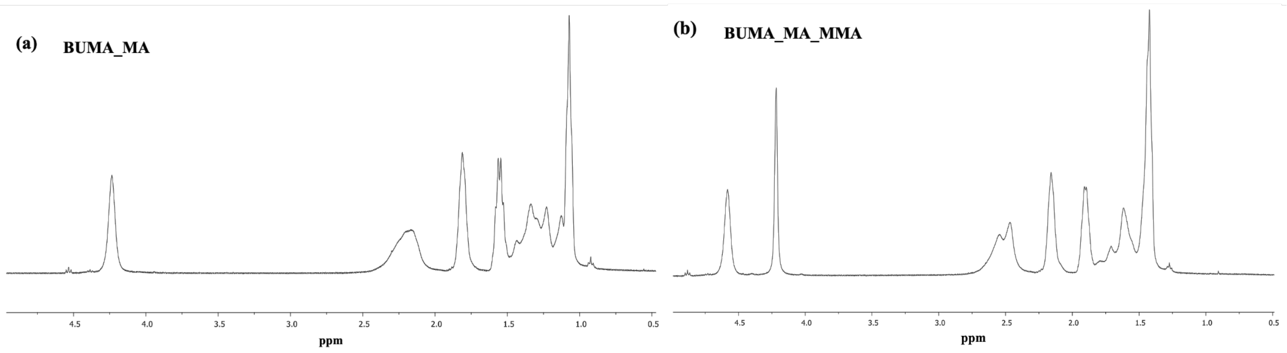

Figure S2. ^{1}H NMR spectrum of (a) BUMA_MA and (b) BUMA_MA_MMA polymers.

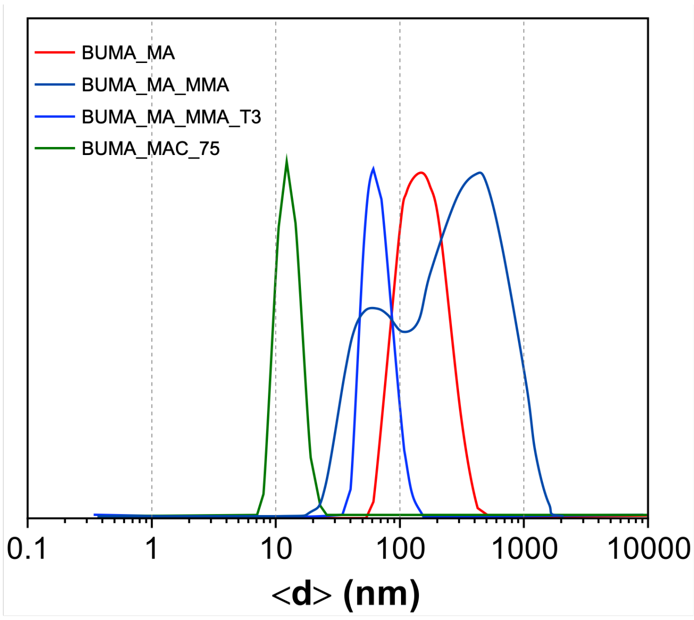

Figure S3. Dynamic light scattering by volume data relative to BUMA_MA, BUMA_MA_MMA, BUMA_MA_MMA_T3 and BUMA_MAC_75 systems.

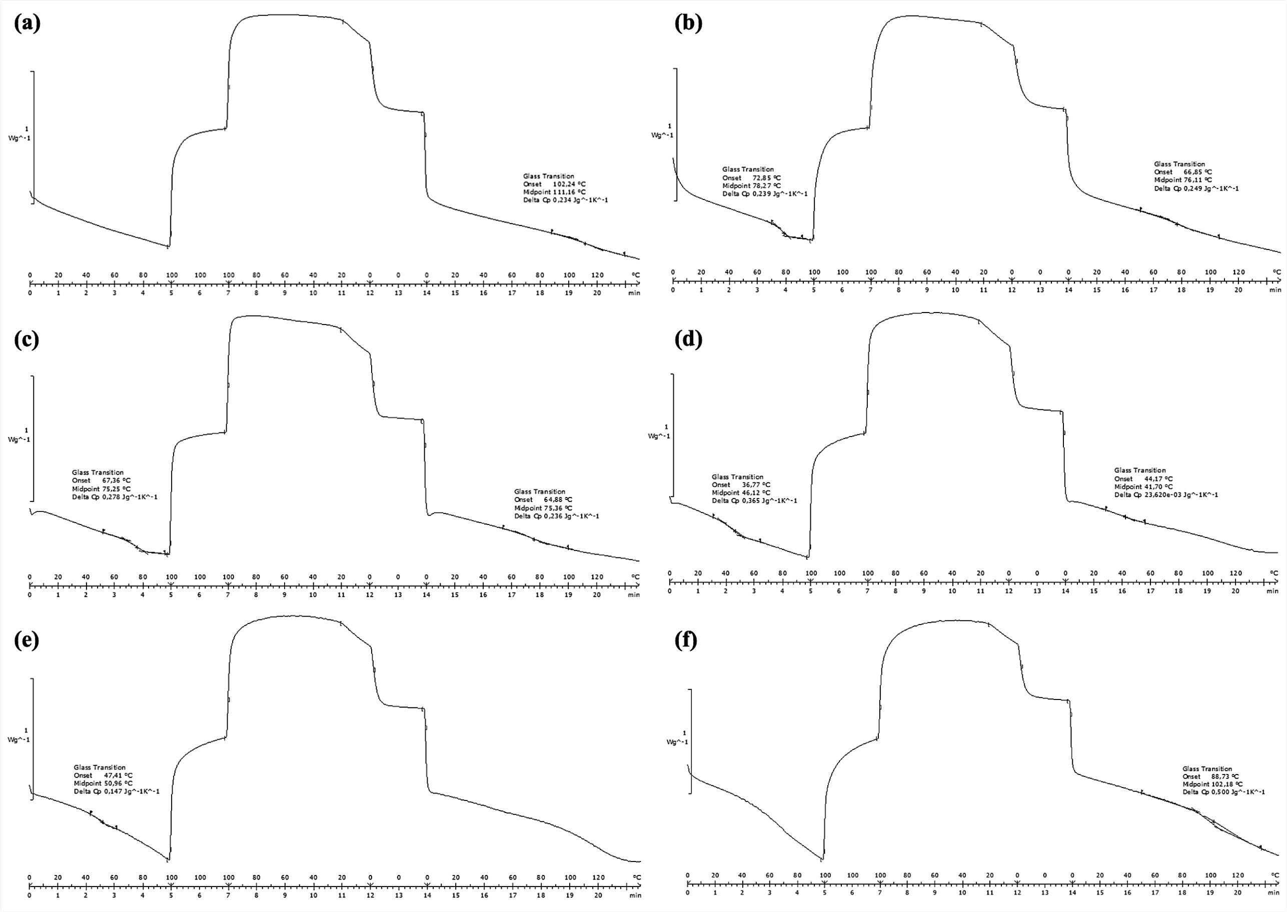

Figure S4. DSC curves relative to (a) BUMA_MA, (b) BUMA_MA_MMA, (c) BUMA_MA_MMA_T3, (d) BUMA_MAC_25, (e) BUMA_MAC_50 and (f) BUMA_MAC_75.
